# The *INT6* Cancer Gene and MEK Signaling Pathways Converge during Zebrafish Development

**DOI:** 10.1371/journal.pone.0000959

**Published:** 2007-09-26

**Authors:** Michal Grzmil, Danny Whiting, John Maule, Corina Anastasaki, James F. Amatruda, Robert N. Kelsh, Chris J. Norbury, E. Elizabeth Patton

**Affiliations:** 1 Sir William Dunn School of Pathology, University of Oxford, Oxford, United Kingdom; 2 MRC Human Genetics Unit and University of Edinburgh Cancer Research Centre, Western General Hospital, Edinburgh, United Kingdom; 3 Departments of Pediatrics, Internal Medicine and Molecular Biology, University of Texas Southwestern Medical Center, Dallas, Texas, United States of America; 4 Centre for Regenerative Medicine, Developmental Biology Programme, Department of Biology and Biochemistry, University of Bath, Bath, United Kingdom; Baylor College of Medicine, United States of America

## Abstract

**Background:**

*Int-6* (integration site 6) was identified as an oncogene in a screen of tumorigenic mouse mammary tumor virus (MMTV) insertions. *INT6* expression is altered in human cancers, but the precise role of disrupted *INT6* in tumorigenesis remains unclear, and an animal model to study *Int-6* physiological function has been lacking.

**Principal Findings:**

Here, we create an *in vivo* model of Int6 function in zebrafish, and through genetic and chemical-genetic approaches implicate Int6 as a tissue-specific modulator of MEK-ERK signaling. We find that Int6 is required for normal expression of MEK1 protein in human cells, and for Erk signaling in zebrafish embryos. Loss of either Int6 or Mek signaling causes defects in craniofacial development, and Int6 and Erk-signaling have overlapping domains of tissue expression.

**Significance:**

Our results provide new insight into the physiological role of vertebrate Int6, and have implications for the treatment of human tumors displaying altered *INT6* expression.

## Introduction

Embryonic development and tumour development often share underlying molecular mechanisms–a concept illustrated by the identification of genes disrupted by the mouse mammary tumor virus (MMTV) in mammary cancers [1]. An important example, the *Int-1* gene which is a common integration site for MMTV in mammary tumours, encodes the homologue of the *Drosophila wingless* gene [2], [3] and was subsequently named *Wnt1* (wingless/Int) in recognition of this conserved function. Wnt signaling is now known to be disrupted in many human tumor types, especially colon cancer [4]. Other *Int* genes, such as *Int-2* and *4* (Fgf3, 4), and *Int-3* (Notch4), encode mitogens and regulators of development that are also misactivated in many cancers [1], [5].

In the majority of cases, MMTV activates *Int* gene expression as a result of proviral integration upstream of the promoter region. Remarkably, all three MMTV insertions found in *Int-6*, which encodes a component of the eukaryotic translation initation factor 3 (eIF3), were found to lie within introns, and in the opposite transcriptional orientation to the *Int-6* gene, creating a truncated *Int-6* mRNA [1], [6]. Ectopic expression of equivalently truncated *Int-6* can transform cell cultures [7], [8], and promote persistent mammary alveolar hyperplasia and tumorigenesis in transgenic mice [9].

Despite important evidence in favor of a role for INT6 in human tumourigenesis [10]–[12], the molecular basis for INT6 in cancer development remains unresolved. Highly conserved in eukaryotes, INT6 contains a PCI domain, found in proteins of the 19S regulatory lid of the proteasome, the COP9 signalosome (CSN), and the eIF3 translation initiation complex; all three complexes share overall structural similarity, and INT6 has been found associated with each [13]. When overexpressed in yeast, Int6 induces multi-drug resistance by activating an AP-1 transcription factor [14], [15], and in human cells, the range of INT6 function includes orderly progression through mitosis [16], regulation of the proteasome-dependent stability of MCM7 [17] and HIF2α [18], and nonsense mediated mRNA decay [19].

With no animal model for Int6 loss-of-function available, we reasoned that an understanding of *INT6* during development would provide novel insight into INT6 function in normal vertebrate cells, thereby providing a new perspective on INT6 function in cancer formation. Here, using zebrafish and mammalian cells, we describe the first Int6 loss-of-function phenotype in an animal, and link Int6 with a signaling pathway, that like those effected by other *Int* genes, is critical for both development and cancer.

## Results

### Int6 is essential for zebrafish embryogenesis

**Figure 1:**
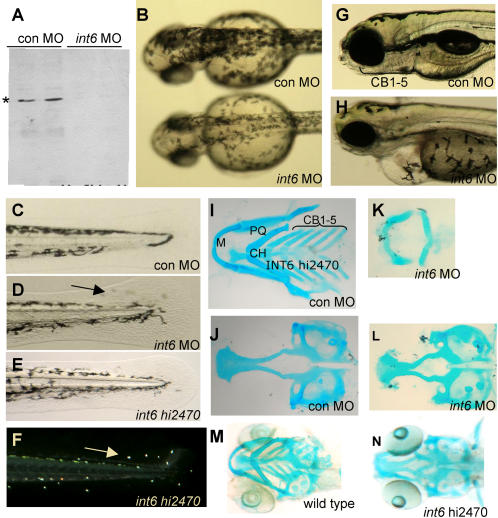
Int6 is essential for zebrafish embryonic development. (A) Western blot analysis of Int6 (*) in zebrafish embryos injected with a control (con) MO or *int6* MO. (B) *int6* morphant melanocytes are less darkly pigmented. (C–F) Int6 is required for pigment cell placement in the tail, as *int6* morphants and *int6^hi2470^* mutants have misplaced pigment cells in the tail fin (D, arrow). Ambient light illuminates the iridophore ‘star-light’ pattern seen in the *int6^hi2470^* embryos (F, arrow). (G–H) By 5 dpf, embryos injected with a con MO have clearly visible certobranical arches, while *int6* morphants do not have visible certobranical arches, in addition to other abnormalities, including unconsumed yolk sac, heart and eye development. (I–N). Alcian blue staining of 5 dpf embryos shows loss of ceratobrancial arches 1 through 5. M, Meckel's; PQ, palatoquadrate; CH, ceratohyal; CB, ceratobrancial.

We chose to study the physiological role of zebrafish Int6 during development, using morpholino oligonucleotides (MOs) to reduce Int6 protein, as well as an *int6^hi2470^* insertional mutant line (kindly provided by N. Hopkins, A. Amsterdam and S. Farrington. M.I.T.). Zebrafish Int6 is over 90% identical in its amino acid sequence to human INT6 (*Ensembl* ENSDARG00000002549) and using an Int6 antibody raised against the N-terminus of the human INT6 [20] we determined that the *int6* MO resulted in loss of Int6 (Figure 1A). As INT6 has been implicated in G2/M-phase cell cycle control, we first performed whole-mount immunohistochemistry with the late G2/M phase marker, phospho-histone H3, and found only slightly reduced numbers of cells in late G2/M phase in the *int6* morphant compared to the control (Figure S1). Importantly, we found that embryos injected with *int6* MO had specific developmental defects (Figure 1B–N), most notably reduced melanisation 2 days post-fertilization (dpf: *int-6* MO n = 51/53; con MO n = 0/35; *int-6 5MM* n = 3/31); misplaced pigment cells in the tail 3 dpf (*int-6* MO n = 46/49; con MO n =  3/30); and abnormal jaw morphogenesis, with cartilage elements reduced or malformed at 4 and 5 dpf (*int-6* MO n = 81/85 4 dpf, n = 76/83 5 dpf; con MO 1/67 4 dpf, n = 1/61 5 dpf). The craniofacial and pigment cell defects observed in the *int-6* morphant and *hi2470* mutant suggest that *int-6* might contribute to development of neural crest-cell (NCC) derivatives. We used multiple markers of NCCs and their derivatives to assess when these phenotypes arise, and found Int6 did not appear to be required for the specification or organization of premigratory and migrating cartilage precursors (Figure S2). In contrast, alcian blue cartilage staining revealed a specific loss of the five ceratobranchial cartilage elements in the *int6* morphants, whereas Meckel's, palatoquadrate, and hyoid cartilage were all present, albeit misshapen (5.5 dpf *int6* MO n = 45/53; con MO n = 1/34; Figure 1I–L, Figure S3). Expression of *int6* mRNA restored normal craniofacial elements to the *int6* morphant (*data not shown*); and the *int6^hi2470^* mutant had an almost identical craniofacial phenotype (Figure 1M, N) indicating a genuine requirement for Int6 in craniofacial development.

### Loss of Int6 alters MEK protein and Erk signaling

**Figure 2:**
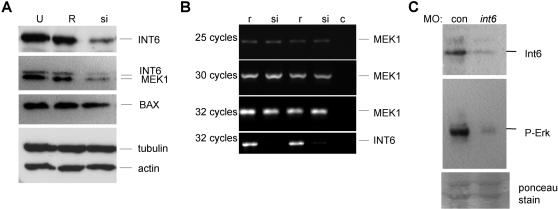
INT6 is required for MEK protein levels and Erk-signaling. A. Osteosarcoma U2-OS cells untransfected (U), or transfected with siRNA targeted against the *INT6* mRNA (si) or the reverse sequence (R), show reduced levels of INT6 and MEK1 protein specifically after transfection with *INT6-*siRNA, but no reduction in BAX, tubulin or actin protein levels. (B) Semi-quantitative-PCR shows *MEK1* mRNA is unaffected in reverse sequence and *INT6*-siRNA treated cells, coupled with the expected reduced levels of the *INT6* message in the *INT6*-siRNA transfected cells. c, PCR control without DNA. (C) Phospho-Erk levels are reduced in *int6* morphants, while ponceau stain detects equal loading of protein on the gel.

Biochemical evidence in fission yeast suggests that Int6 is part of a specialized eIF3 translation initiation complex that may target specific mRNAs for translation [21]. Given the involvement of INT6 in cell proliferation [16], western blots using a panel of antibodies against proteins involved in the cell cycle and associated signalling pathways were performed using lysates from control and INT6 siRNA transfected MDA-MB-231 cells. Of 16 proteins investigated in this way, only MEK1 levels were altered by INT6 siRNA transfection (Figure 2). As previously reported [20], we found *INT6*-siRNA cell lysates had reduced levels of INT6 protein compared with the untransfected and reverse *INT6*-siRNA sequence. We also found a dramatic reduction of MEK1 protein levels that correlated with loss of INT6, while BAX, tubulin and actin protein levels appeared unaffected in the *INT6*-siRNA transfected cells (Figure 2A). The loss of MEK1 was specifically at the protein level, as semi-quantitative-PCR showed normal levels of *MEK1* mRNA in *INT6*-siRNA treated cells, as well as the expected reduced levels of the INT6 message in the *INT6*-siRNA transfected cells (Figure 2B). The possibility that *INT6* may affect MAPK signaling through control of MEK protein levels prompted us to examine the phosphorylation state of Erk1/2, downstream targets of the Mek kinases, in *int6* morphant zebrafish embryos. Compared with control MO embryos, *int6* morphant embryo lysates had reduced phospho-Erk levels (Figure 2C). These data suggest a novel function for Int6 in the control of MAPK signaling in the developing embryo, possibly by direct control of MEK1 protein levels.

### Int6 and Mek pathways converge during development

**Figure 3:**
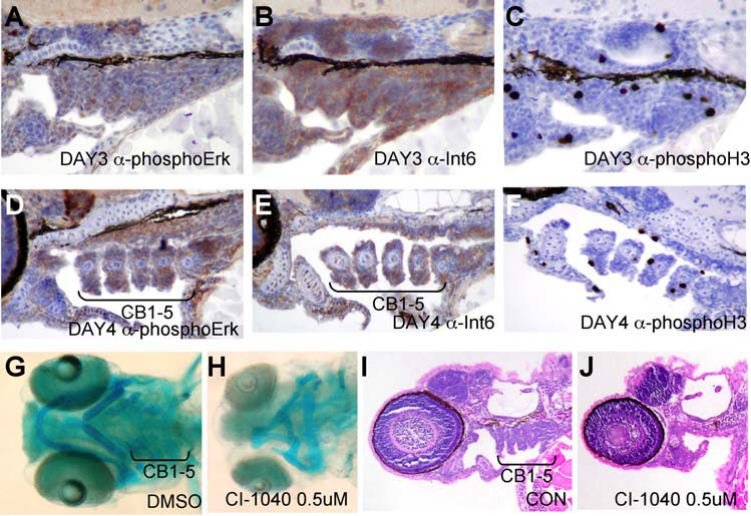
Int6 and phospho-Erk expression in the developing zebrafish embryo, and pharmacological inhibition of Mek alters ceratobrancial (CB) arches. (A–F). Immuno-histochemistry of Int6 and phospho-Erk in the developing craniofacial tissues, counter stained with hematoxylin, and phospho-histone H3 to show cycling cells. (G, H) Ventral whole mount views of Alcian blue stained pharyngeal cartilages show loss of ceratobrancial arches 1–5 and a reduction of Meckel's (M), palatoquadrate (PQ) and ceratohyal (CH) cartilages in 4 dpf embryos treated with 0.5 uM CI-1040. (I, J) Sections of 4 dpf embryos hematoxylin and eosin stained after 0.5 uM CI-1040 treatment reveals loss of CB arches 1–5 (brackets). E, Ethmoid plate; PC, Parachordal cartilage.

**Figure 4:**
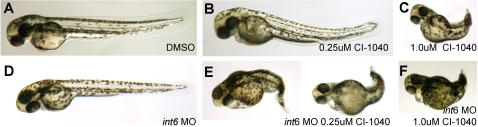
Int6 and Mek signaling interact *in vivo*. (A–C). Only embryos treated with the 1.0 µM CI-1040, and not 0.25 µM, show a loss of posterior structures, in contrast to (D) *int6* morphants (*int6* MO 0.25 ng). (E, F) In combination with 0.25 µM of CI-1040, 0.25 µg of *int6* MO causes a dramatic alteration of the anterior-posterior axis.

If Int6 controls Mek activity in the developing embryo, we theorized that specific developing tissues might have overlapping expression domains of Int6 protein and phospho-Erk activity. Indeed, immunohistochemistry with antibodies directed against Int6 and phospho-Erk revealed overlapping domains of expression in the developing craniofacial region in 3 and 4 dpf embryos (Figure 3A–F). Strong Int6 tissue-specific expression was also detected in the developing intestine and lens, regions that had little or no phospho-Erk expression (Figure S4). Given the observed phospho-Erk and Int6 expression in the craniofacial region, we hypothesized that some of the Int6 phenotypes, such as the jaw formation defect, might be phenocopied by repression of Erk signaling. As interpretation of MO phenotypes has recently been complicated by the identification of MO-induced p53-dependent craniofacial defects [22], we used an alternative approach – the highly selective, clinically active MEK inhibitor CI-1040 [23] – to reduce Mek signaling in zebrafish. We added the drug at 4 hpf at a concentration of 0.25, 0.5, and 1.0 µM, and confirmed loss of phospho-Erk expression by Western blot analysis (*data not shown*). Notably, the addition of CI-1040 caused a dose-dependent loss of the posterior structures of the embryo, such that 1.0 µM CI-1040 caused a severe anterior-posterior (AP) axis defect (Figure 4A–C), consistent with a role for FGF signaling in the development of the AP axis [24]. CI-1040 also caused loss of ceratobranchial cartilage elements, while the anterior elements – Meckel's, palatoquadrate and hyoid cartilages – were present but misshapen (Figure 3G–J), similar to the effects seen in *int6* morphants and mutants.

To further elucidate the biological relevance of Int6 and Mek signaling, we took advantage of the ease with which signaling pathways can be altered pharmacologically in specific genetic contexts in the zebrafish system. We reasoned that if Int6 contributes to activation of Mek signaling, then embryos with reduced Int6 should be hypersensitive to low doses of the MEK inhibitor CI-1040. In control embryos treated with 0.25 µM CI-1040, no changes in the anterior-posterior axis were detected (Figure 4B). In addition, *int6* morphants generated by low doses of MO (0.25 ng) did not have an altered AP axis (Figure 4D). In contrast, in combination with low doses of CI-1040, the low dose *int6* morphant showed a severely enhanced AP axis phenotype (Figure 4E). Taken together, these data provide further evidence that *int6* may play a role in modulating MEK signaling *in vivo*.

## Discussion

Activated in most cancers, the MAPK signaling pathway is among the most attractive targets for novel anti-cancer therapies [23]. Like MAPK signaling pathways, most of the *Int* pathways - Wnt, Fgf, and Notch - are conserved regulators of development that are frequently activated to promote oncogenesis. We provide evidence that, like other *Int* gene products, *Int6* is required for vertebrate development (Figure 1), in part by providing a novel layer of MAPK signal transduction regulation (Figure 2). With the wide range of cellular activities attributed to INT6, the mechanistic detail of this control remains to be understood; our early investigations indicate reduction of MEK1 in *INT6*-siRNA treated mammalian cells is not dependent on the proteasome (M.G. & C.J.N., *unpublished data*), making direct MEK1 regulation by INT6-dependent translation a possibility.

Recently, importance of RAS, RAF and MEK in human disease has been extended beyond cancer by the discovery that human germ-line mutations in these genes cause the LEOPARD-Noonan family of syndromes [25]. Detailed immunohistochemical studies in mice have identified highly regulated, specific domains of discrete and dynamic ERK phosphorylation throughout development, including the pharyngeal arches and limb buds [26]. In the first Int6 protein expression studies in a whole developing animal, we show that Int6 has regionally overlapping domains of protein expression with phospho-Erk, primarily in the craniofacial region (Figure 3, Figure S4). Lending biological significance to these observations, we show that phenotypic characteristics are shared between the loss of Int6 and inhibition of Mek activity (Figure 3). In addition, partial loss of Int6 causes embryos to be highly sensitive to a mildly compromising dose of Mek inhibition, revealing an *in vivo* interaction between Int6 protein expression and developmental Mek-Erk signaling (Figure 4). As early over-expression of Ras-Raf-Mek signaling causes morphologic defects, we are currently generating transgenic lines that allow temporal control of Mek signaling, which will be a valuable tool for deeper genetic dissection of the Int6-Mek-Erk relationship *in vivo*. It will be critical in future studies to establish if Int6 is capable of controlling both Mek1 and Mek2; our initial MO studies indicate that MEK2 may have a specific role in melanocyte migration (C.A. & E.E.P, *unpublished data*), raising the possibility that the pigment cell migration defects observed in the *int6* morphants also reflect altered MEK signaling.

FGF signaling is crucial for skeletal development, exemplified by the mutations that disrupt FGF signaling in human genetic skeletal abnormality syndromes [27]. In the developing mouse embryo, most phospho-ERK domains overlap with FGF signaling domains [26]. FGF signaling molecules are candidates for upstream activation of the Int6-moderated Mek-Erk signaling that shapes the craniofacial skeleton in vertebrates [28], [29], and candidate downstream targets of Int6-Mek-Erk signaling include the chondrocyte differentiation transcription factor Sox9, which requires Mek activity for transcriptional activity [30]. We also note that *erk2*, but not *erk1*, is specifically expressed in the pharyngeal arches in two-day old zebrafish embryos [31], possibly suggesting that Int6-Mek modulation in the developing craniofacial region may specifically signal through targets of Erk2.

Relating the Int6 modulation of Mek-Erk signaling to cancer development is a new angle for future investigation. One possibility is that in MMTV induced mammary tumors, the truncated Int-6 protein may act as an oncogene by altering MEK-ERK signaling. We propose that the diverse cellular locations of Int6, combined with the temporal expression and localization of Mek1/2 and Erk1/2, may result in fine-tuning of Mek-Erk signaling pathways in specific tissues during development, and may have important implications for the role of INT6 in tumorigenesis.

## Methods

### Zebrafish husbandry and morpholino studies

Zerbafish (*Danio rerio*) lines AB, AB*, and AB*-TPL were raised and staged as described [32], [33]. MOs (Table 1) were designed by and purchased from Gene Tools, LLC (USA), and 1 ng injected into one-cell stage embryos.

**Table 1:** Oligonucleotides used in this study

*Method*	*Symbol*	*Oligonucleotide*
*Morpholino*		
Control	*con* MO	5′ CCTCTTACCTCAGTTACAATTTATA
*int6* Translation block	*int-6* MO	5′ GGTCAGATCGTACTCCGCCATGATG
*int6* 5-base pair mismatch	*int-6* 5MM	5′ GGTgAGATCcTAgTCCGCgATcATG
*siRNA*		
*INT6* sense siRNA (si)	*INT6*-siRNA	5′ GAACCACAGUGGUUGCACAUU
*INT6* reverse siRNA (R)	R	5′ UUACACGUUGGUGACACCAAG
*RT-PCR primers*		
*MEK1* forward		5′ ATTATTGTTCCCCTAAGTGGATTG
*MEK1* reverse		5′ TTACAACAGCATTGGTACTTGGAT
*INT6* forward		5′ ATGGCGGAGTACGACTTGACT
*INT6* reverse		5′ TCAGTAGAAGCCAGAATCTTGAGT
*Actin* forward		5′ CGTGATGGTGGGCATGGGTCA
*Actin* reverse		5′ CTTAATGTCACGCACGATTTCC

### Phenotype analysis

Phenotype analysis were performed as described: cell cycle studies [34]; alcian blue staining [35]; probe synthesis and whole-mount in-situ hybridizations [36]. cDNA probes for neural crest markers were the kind gift of David Raible (University of Washington, USA). Polyadenylated *int6* mRNA was generated using Ambion mMessage mMachine (#1340).

### Cell culture and RT-PCR analysis

MDA-MB-231 cells were grown and transfected as described [19] using Lipofectamine (Invitrogen) with si-oligonucleotides (Table 1; Eurogentec) at a final concentration of 100 nM in Optimem (Gibco). Forty-eight hours after transfection total RNA was isolated (RNeasy Mini Kit; Qiagen) and one-step RT-PCR reactions (Qiagen) accomplished using specific primers (Table 1).

### Immunoblotting

Whole-cell lysates and zebrafish extracts were generated [31], [19] and immunohistochemistry was performed as described [36]. Antibodies used as in Table 2.

**Table 2:** Primary antibodies used in this study

*Antibody*	*Source*	*Working dilution*
anti-Phospho-Histone H3 (ser 10) #9706	Cell Signaling Technology	1∶1000 western blot 1∶100 immunohistochemistry
anti-Phospho-p44/42 MAPK #9160	Cell Signaling Technology	1∶1000 western blot 1∶100 immunohistochemistry
anti-Int6 CN24	Watkins and Norbury, 2004	1∶500 western blot 1∶50 immunohistochemistry
anti-α-tubulin	K. Gull, Oxford	1∶5000 western blot
anti-BAX N-20	Santa Cruz Biotechnology	1∶1000 western blot
anti-MEK1 H-8	Santa Cruz Biotechnology	1∶2000 western blot
anti-actin N-20	Sigma	1∶4000 western blot

## Supporting Information

Figure S1 Cell cycle analysis of int6 morphants. Whole-mount immunohistochemistry with the late G2/M phase marker, phospho-histone H3 shows only slightly reduced numbers of cells in late G2/M phase in the int6 morphant compared to the control. Similarly, DNA content as measured by flow cytometry reveals only a slight reduction of cells in G2/M phase in the int6 morphant. Thus, we find that loss of Int6 in normal vertebrate cells (as well as in additional human cancer cell lines, M.G. & C.J.N. unpublished data) does not appear result in an accumulation of cells in G2/M progression.
(4.75 MB TIF)

Figure S2 Lateral views of in situ hybridization of neural crest markers in control and int6 morphants, revealing no change in cell number or migration as indicated by the apparently normal expression of dlx2 (stages 6–36 hpf, examined at two hour intervals), nor of early markers of NCC and melanocytes, such as sox10, crestin, snail and mitfa (24 hpf) in int6 morphants. These observations were extended by examination of a transgenic sox10-GFP line (1) revealing unaltered GFP-expressing NC-derived cells in int6 morphants within the first 48 hpf, but a loss of GFP expressing differentiated pharyngeal arches 3–7 by 3 dpf (data not shown).
(40.41 MB TIF)

Figure S3 Development of the pharyngeal arches in the developing control (A–C) and (D–F) int6 morphant animals. Note the loss of pharyngeal arches (A, bracket) in the int6 morphants (bracket). Sections were stained with methylene blue. Anterior to the left.
(14.09 MB TIF)

Figure S4 Immunohistochemistry of Int6 and phospho-Erk staining in 4 dpf embryos. (A, B) While Int6 and phospho-Erk signaling overlap in the craniofacial region, they also have distinct patterns, for example in the eye and (C, D) gut. We note that while Int6 and phospho-Erk have overlapping domains of expression in the craniofacial region, Int6 staining in the craniofacial region was stronger than phospho-Erk, and phospho-Erk staining was limited to specific tissues within the craniofacial region. M, Meckel's; E: Ethmoid plate; CH, ceratohyal; CB, ceratobrancial. Sagittal section, anterior to the left.
(17.60 MB TIF)
