# Supplementary figures and images for: The INT6 Cancer Gene and MEK Signaling Pathways Converge during Zebrafish Development
Source: PLoS One. 2007 Sep 26;2(9):e959. doi: 10.1371/journal.pone.0000959 (PMC1978538; doi:10.1371/journal.pone.0000959)

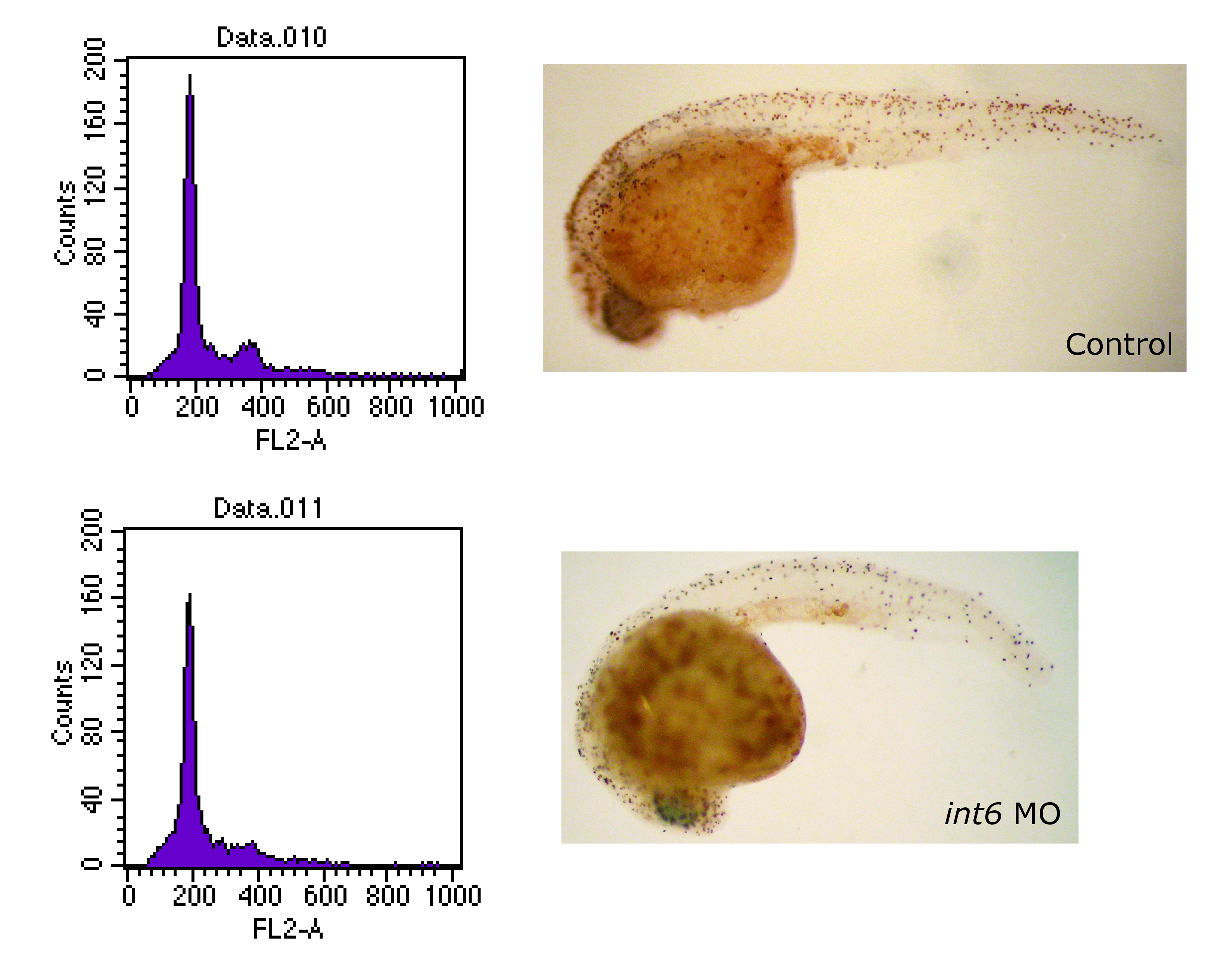

Supplement: Figure S1 — Cell cycle analysis of int6 morphants. Whole-mount immunohistochemistry with the late G2/M phase marker, phospho-histone H3 shows only slightly reduced numbers of cells in late G2/M phase in the int6 morphant compared to the control. Similarly, DNA content as measured by flow cytometry reveals only a slight reduction of cells in G2/M phase in the int6 morphant. Thus, we find that loss of Int6 in normal vertebrate cells (as well as in additional human cancer cell lines, M.G. & C.J.N. unpublished data) does not appear result in an accumulation of cells in G2/M progression. (4.75 MB TIF) [file pone.0000959.s001.tif]

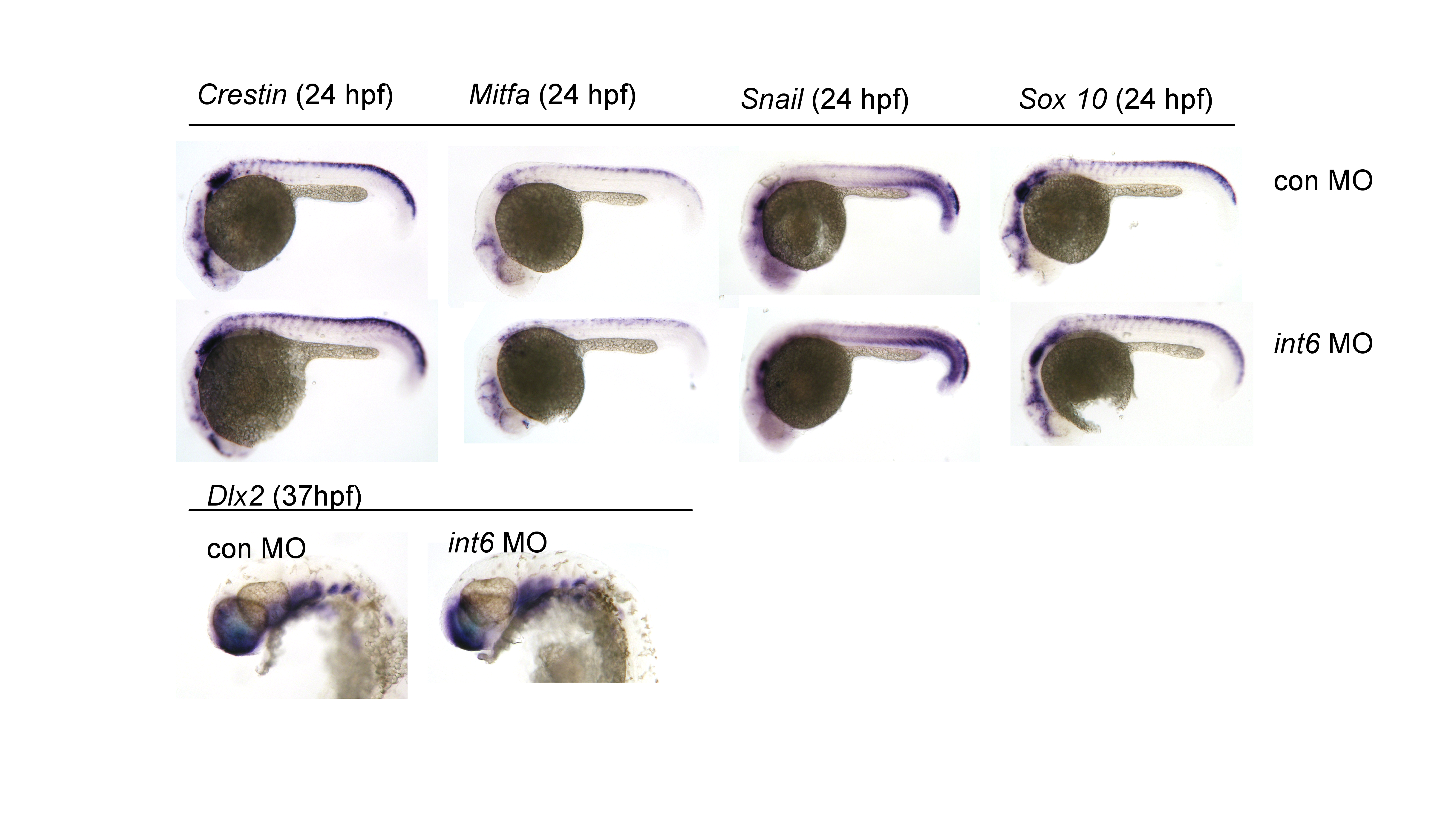

Supplement: Figure S2 — Lateral views of in situ hybridization of neural crest markers in control and int6 morphants, revealing no change in cell number or migration as indicated by the apparently normal expression of dlx2 (stages 6–36 hpf, examined at two hour intervals), nor of early markers of NCC and melanocytes, such as sox10, crestin, snail and mitfa (24 hpf) in int6 morphants. These observations were extended by examination of a transgenic sox10-GFP line (1) revealing unaltered GFP-expressing NC-derived cells in int6 morphants within the first 48 hpf, but a loss of GFP expressing differentiated pharyngeal arches 3–7 by 3 dpf (data not shown). (40.41 MB TIF) [file pone.0000959.s002.tif]

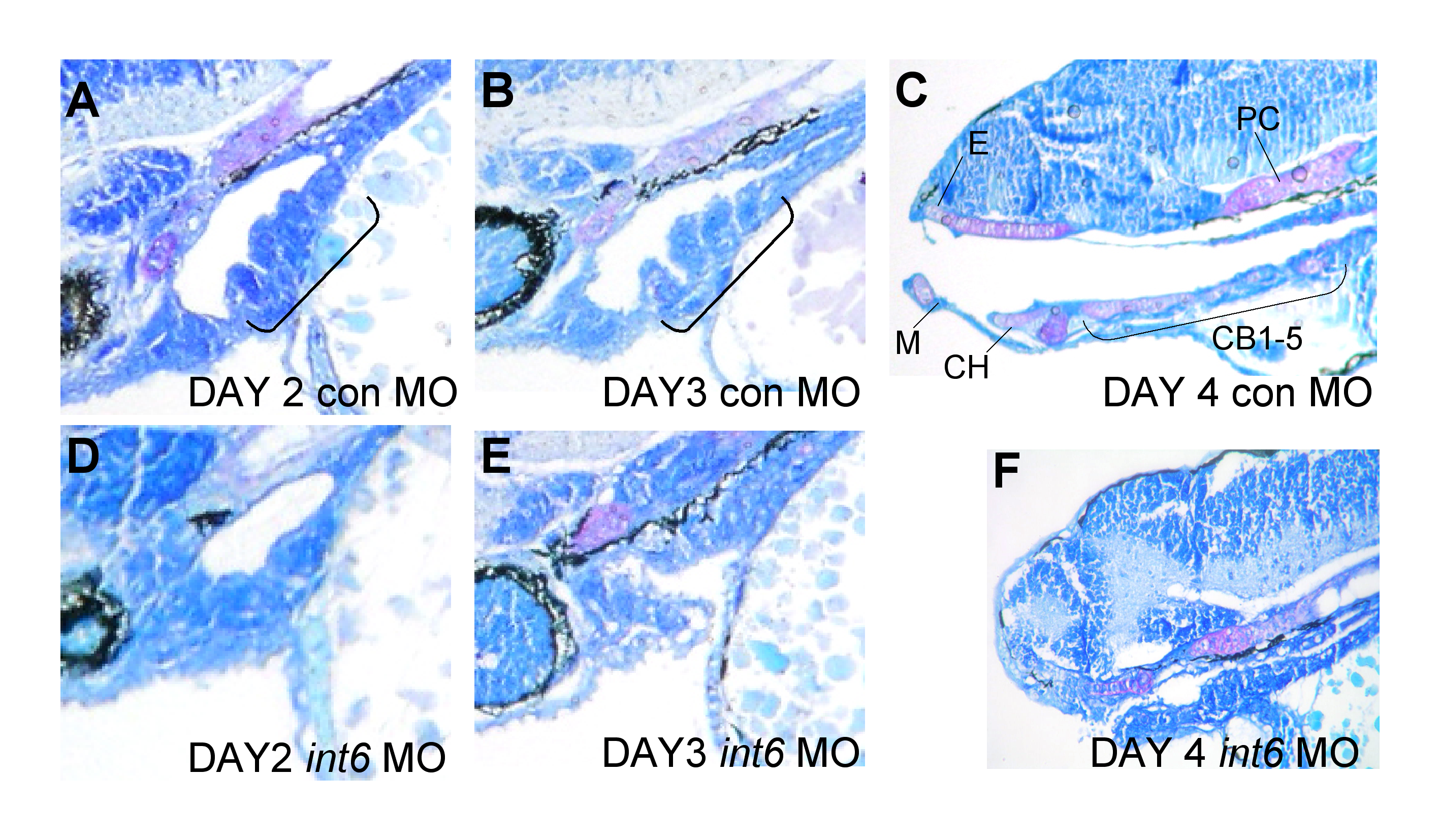

Supplement: Figure S3 — Development of the pharyngeal arches in the developing control (A–C) and (D–F) int6 morphant animals. Note the loss of pharyngeal arches (A, bracket) in the int6 morphants (bracket). Sections were stained with methylene blue. Anterior to the left. (14.09 MB TIF) [file pone.0000959.s003.tif]

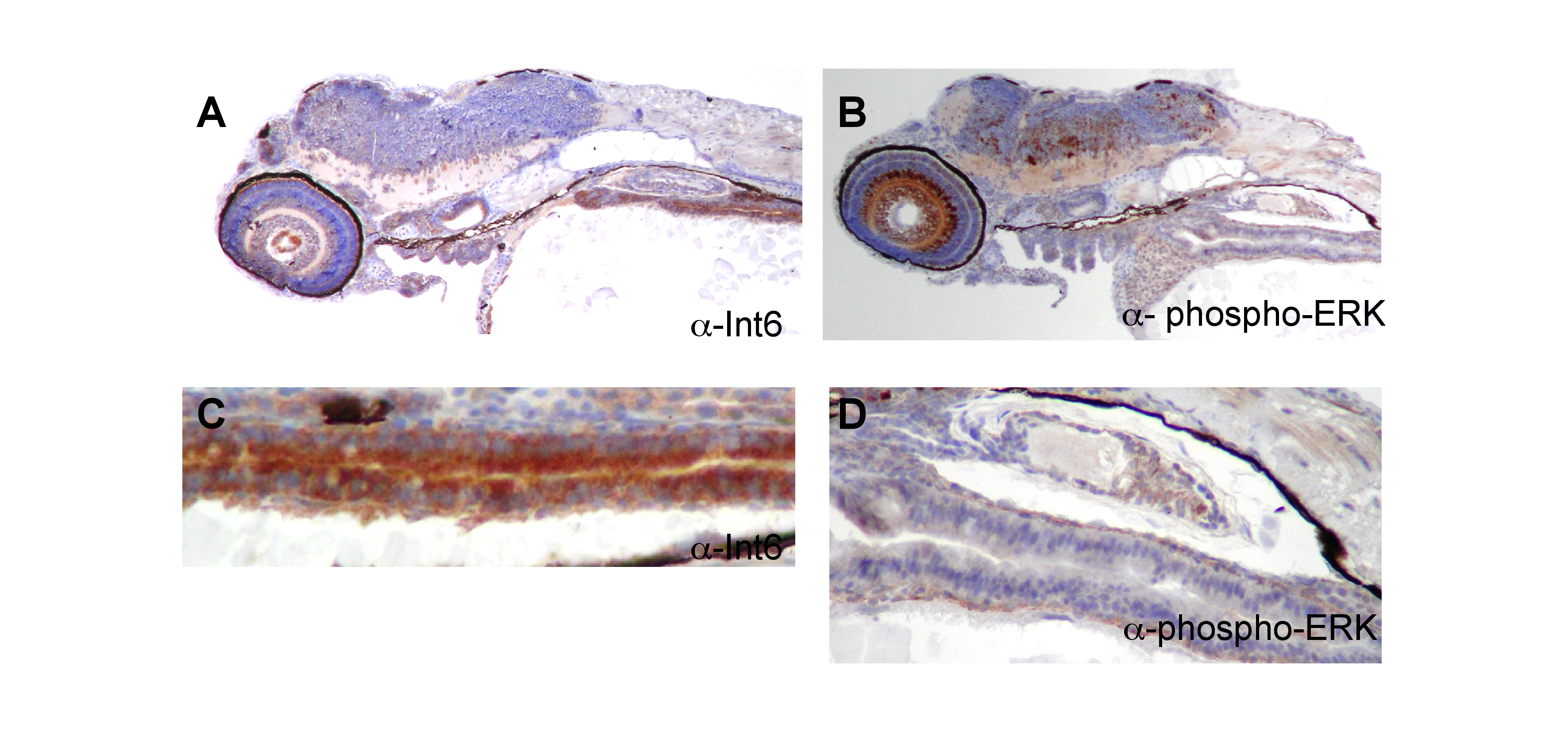

Supplement: Figure S4 — Immunohistochemistry of Int6 and phospho-Erk staining in 4 dpf embryos. (A, B) While Int6 and phospho-Erk signaling overlap in the craniofacial region, they also have distinct patterns, for example in the eye and (C, D) gut. We note that while Int6 and phospho-Erk have overlapping domains of expression in the craniofacial region, Int6 staining in the craniofacial region was stronger than phospho-Erk, and phospho-Erk staining was limited to specific tissues within the craniofacial region. M, Meckel's; E: Ethmoid plate; CH, ceratohyal; CB, ceratobrancial. Sagittal section, anterior to the left. (17.60 MB TIF) [file pone.0000959.s004.tif]
